# Metacognitive therapy (MCT+) in patients with psychosis not receiving antipsychotic medication: A case study

**DOI:** 10.3389/fpsyg.2015.00967

**Published:** 2015-07-09

**Authors:** Ryan P. Balzan, Cherrie Galletly

**Affiliations:** ^1^School of Psychology, Flinders UniversityAdelaide, SA, Australia; ^2^Discipline of Psychiatry, University of AdelaideAdelaide, SA, Australia

**Keywords:** schizophrenia, psychotherapy, delusions, cognitive bias, CBT

## Abstract

**Background:** Psychotherapies for psychosis typically aim to develop an awareness of the implausible content of a delusion or target the underlying *cognitive biases* (i.e., problematic thinking styles, such as hasty decisions and illusory control) that foster and maintain delusional beliefs. A recently designed individual-based treatment entitled *metacognitive therapy* (MCT+) combines these two approaches. Emerging evidence suggests individualized MCT+, when used concurrently with antipsychotic medication, may be an effective psychological treatment for reducing delusional symptoms. However, it remains to be tested whether MCT+ can be effective in patients with active delusions who are not currently receiving psychotropic drugs.

**Method:** We present two cases (one patient with schizophrenia and the other with delusional disorder) experiencing active delusions who underwent 4-weeks of intensive MCT+, without concurrent antipsychotic medication (minimum 6-months unmedicated). Baseline and 6-week follow-up data are presented on a variety of measures assessing delusion symptom severity (i.e., PANSS, PSYRATS, SAPS), clinical insight, and cognitive bias propensity.

**Results:** After 4-weeks of MCT+, both patients showed substantial reduction in delusional symptoms, reported improved clinical insight, and were less prone to making illusory correlations.

**Conclusions:** The presented case studies provide preliminary evidence for the feasibility of MCT+ in treating patients not taking, or resistant to, antipsychotic medication.

## Introduction

Antipsychotic medications are an effective treatment for the symptoms of psychosis, such as delusions and hallucinations, and provide relief for many people with psychotic disorders. However, many studies report that 20–30% of clients with psychosis do not respond to these medications (Tandon, 2011; Leucht et al., 2012). Even when these treatments are effective, they are often associated with only medium effect sizes relative to placebo, high levels of relapse, issues with insight, and adherence, and serious side-effects (e.g., Leucht et al., 2009; Muench and Hamer, 2010). Accordingly, interest in adjunctive non-pharmacological treatments has gathered momentum in recent years. For example, cognitive-behavioral therapy for psychosis (CBTp) is now routinely administered alongside antipsychotic medications to treat the core symptoms of psychosis (Lecomte et al., 2008; Bechdolf et al., 2010; Morrison et al., 2014). CBTp aims to identify and actively modify maladaptive delusional beliefs, attitudes and behaviors often associated with psychosis, and thereby helps clients to become aware of alternative explanations and coping strategies (Steel, 2013). Reviews and meta-analyses of its efficacy as an adjunct therapy to pharmacological treatments have shown that CBTp adds small to medium effect sizes on top of medication, and may represent an effective treatment alternative for medication-resistant or non-adherent clients (Wykes et al., 2008; Farhall and Thomas, 2013; Huhn et al., 2014).

Built on the principles of CBTp, novel psychological interventions for treating delusions are now starting to focus on the underlying cognitive and social biases that contribute to the formation and maintenance of delusional beliefs (Bell et al., 2006; Balzan et al., 2012, 2013a,b; Garety and Freeman, 2013), rather than targeting the idiosyncratic delusions specific to the individual client (Moritz et al., 2010b). One such intervention is metacognitive training (MCT), which is a group-based program consisting of eight intervention sessions (available free of charge from www.uke.de/mkt). MCT is categorized under six cognitive and social biases (i.e., attribution biases, jumping to conclusions, belief inflexibility, overconfidence in errors, theory of mind deficits, and depressive cognitive schemata). The program attempts to raise the metacognitive awareness of such biases within clients, thereby planting the “seeds of doubt,” encouraging critical reflection, and ultimately reducing the severity of delusional symptoms. Similar to CBTp, clinical trials have consistently shown that MCT is effective in reducing delusional symptoms relative to controls (Aghotor et al., 2010; Moritz et al., 2011a, 2014a,b; Favrod et al., 2013), and exerts *sustained* effects on the reduction of delusions over and above the effects of antipsychotic medication (for an in-depth summary and review of MCT see Balzan et al., 2014b; Moritz et al., 2014c).

In response to the emerging efficacy for the group-orientated MCT program, an individually administered program entitled “metacognitive therapy” (MCT+), has recently been developed. This program combines the “process-oriented” approach of the MCT group-training with elements of individual cognitive-behavioral therapy for psychosis (CBTp). The combined approach involves relating information from the original MCT modules to the individual experiences, observations, and symptoms of the individual client (Moritz et al., 2010a). MCT+ comprises a similar layout to the group-based MCT, and covers the same six cognitive and social biases. However, the individualized approach also includes opportunities for clients to share their own personal experiences in relation to the material being presented. This allows for a greater range of therapeutic strategies, such as establishing therapy goals (e.g., reducing paranoia in public spaces), reality testing (e.g., recalling certain events in everyday life that could be regarded as clear evidence for delusional ideas), and Socratic discussion (i.e., extensive questioning to generate pros/cons and consequences of a particular viewpoint). To date, the evidence for MCT+ is limited to two small-scale studies (Moritz et al., 2011b; Balzan et al., 2014a) and a single case report (Vitzthum et al., 2014), which suggest that the therapy program is effective in significantly reducing delusion severity and conviction, increasing clinical insight, and improving performance on cognitive bias tasks.

Despite the demonstrated efficacy of CBTp, MCT, and MCT+ in alleviating the symptoms of psychosis, few trials have been able to test the efficacy of these psychotherapies in the absence of antipsychotic medication. This is an important clinical issue, as people with psychosis may become non-adherent and discontinue taking antipsychotic treatment, or demonstrate treatment resistance to these medications (Lieberman et al., 2005). While at least one trial has been able to show the efficacy of cognitive therapy in reducing psychiatric symptoms in people with schizophrenia spectrum disorders who had chosen not to take antipsychotic drugs (Morrison et al., 2014), more reports are required. The purpose of the current paper is to detail the case histories of two clients with psychosis (one with schizophrenia and one with delusional disorder), neither of whom were taking antipsychotic medication, but both had received 4-weeks of MCT+ as part of a larger randomized control trial investigating the effectiveness of MCT+ in reducing the symptoms of psychosis. MCT+ is a useful platform in order to observe the efficacy of psychotherapies in the absence of pharmacological treatment as it combines the approaches of both CBTp and group-lead MCT, and therefore may be more effective in reducing delusional symptoms than either treatment offered in isolation.

## Background

The following case study deals with two clients diagnosed with a psychotic disorder, and who were experiencing active delusions at the time of entering a larger randomized control trial investigating the effectiveness of MCT+. Neither client was taking antipsychotic medication (or any other psychotropic), or receiving any other psychological therapy, at the time of investigation (6-months unmedicated for Client 1; 9-months unmedicated for Client 2), and both were outpatients living in the community (for full summary of baseline symptoms, see Table 1). Clinical insight was minimal for both clients.

**Table 1:** **Raw baseline and follow-up scores of symptom severity (PANSS; PSYRATS; SAPS; PDI-21), insight (SAI), and cognitive bias performance (illusory control) for both clients**.

	**Client 1 (schizophrenia)**		**Client 2 (delusional disorder)**	
	**Baseline**	**Follow-up**	**Baseline**	**Follow-up**
**SYMPTOM SEVERITY**				
PANSS (total)	89	80	65	46
*PANSS-Delusions (P1)*	6	5	5	3
*PANSS-Positive*	26	21	18	11
*PANSS-Negative*	16	15	17	9
*PANSS-General*	47	44	30	26
PSYRATS-Hallucinations	34	28	–	–
PSYRATS-Delusions	23	20	18	12
SAPS-Hallucinations	23	19	–	–
SAPS- Delusions	37	30	14	9
PDI-21-Global	281	243	83	58
*PDI-21-Distress*	77	74	23	17
*PDI-Preoccupation*	89	77	27	19
*PDI-Conviction*	95	75	25	15
**INSIGHT**				
SAI	2	7	8	10
**COGNITIVE BIAS TASK**				
Illusory control (%)	50	37.5	37.5	0
Illusory control: perceived connection (%)	50	37.5	50	12.5

*PANSS, Positive and Negative Syndrome Scale; PSYRATS, Psychotic Symptom Rating Scales; SAPS, Scale for the Assessment of Positive Symptoms; PDI-21, 21-item Peters et al Delusions Inventory; SAI, Schedule for Assessing Insight (higher scores indicate greater insight; max score = 18); illusory control task was completely non-contingent (i.e., any perceived control >0 was interpreted as “illusory control”)*.

### Client 1

At the time of his involvement in the trial, Client 1 was a 20-year-old male who had been diagnosed with treatment-resistant schizophrenia (limited success on trials of olanzapine, ziprasidone, risperidone (oral and depot), pericyazine, and quetiapine). He was diagnosed from a young age (records indicate first diagnosis of schizophrenia was made at the age of 14-years), with four psychiatric hospital admissions. He was unemployed (receiving a government youth allowance) and was living with a young family (two adults, two children under 5-years) who had taken him in. He would otherwise have been homeless. Pre-morbid IQ was estimated at 86 (using the WTAR; see Design below).

Psychiatric symptoms included vivid auditory hallucinations, which he described as a number of male and female voices that (i) commented on what he was doing or thinking, (ii) made derogatory comments, and (iii) commanded him to harm himself and others. He did not feel compelled to act on the command hallucinations. He also experienced visual hallucinations, including seeing floating facial parts in the dark. His delusional beliefs included being persecuted by government agencies who had been tracking him via secret cameras on his street (which had recently increased in number), and a device planted in his neck; he often had thoughts removing the device, but worried about cutting his neck. He was a long-term frequent user of THC (“1–2 bags per day” since he was 12 years old), and was a heavy drinker (consuming approximately “10 liters of wine and a carton of beer” weekly). Aggression, highly-impulsive behavior (e.g., assaulting strangers), and psychiatric symptoms intensified whilst taking either substance. A risk assessment identified some occasional suicidal thoughts, but overall low risk, with no definite motive or detailed plans. His treatment goals included reducing his paranoia (e.g., leave the house at different times) and conflicts with other people in his street (e.g., no longer accusing them of persecution).

### Client 2

Upon commencing the trial, Client 2 was 31-year-old male, with delusional disorder (diagnosed 2 years prior), unemployed and living on a disability support pension in a private boarding house. He had at least two prior psychiatric hospital admissions. Client 2 had no history of auditory or visual hallucinations, did not describe any current hallucinations, and did not drink alcohol or use THC. Whilst very functional across a number of cognitive domains, with above average intelligence (pre-morbid IQ was estimated at 109), his paranoid delusional ideas have prevented him from obtaining a stable career path, and reaching full social independence.

His core paranoid delusion was that his personal identity and details of his private life were readily available for people to observe. His feeling of being “watched” first arose whilst working in a large warehouse of approximately 400 employees, where he suspected that he was being laughed at and talked about behind his back. These ideas culminated with him confronting the other employees about their knowledge of his personal life, and his subsequent dismissal, whereby he moved across country and relocated to another city in the hopes of escaping the persecution. However, similar persecutory ideas persisted in his new residence, with frequent thoughts that strangers were trying to mess with his mind through social media and the internet, but escalating to the belief that all computers were monitoring and recording his actions and thoughts. He had previously sought help from a psychotherapist with limited success, and hoped the MCT+ sessions could help him to improve his ability to “test reality” and thereby reduce his paranoia and ideas of persecution in social settings.

## Design

Both clients were randomized into the MCT+ treatment group as part of a larger treatment trial that allocated participants to either MCT+ or to cognitive remediation (active control condition). The trial consisted of six sessions, consisting of baseline assessment, four MCT+ sessions (covering all six cognitive biases plus additional material), and a follow-up session that mirrored the baseline measures, which was administered 1-week after completing MCT+ (i.e., 1 month from commencing the trial). Each of these six sessions lasted approximately 90–120 min. Clinical ethics was approved by the Human Ethics Research Committee (TQEH/LMH/MH), Adelaide, Australia.

### MCT+

Following the first baseline session, both clients commenced 4 weeks of MCT+, with one 90–120 min session per week, usually consisting of two MCT+ “units” per session. MCT+ consists of ten units. Unit 1 is designed to build up the therapeutic alliance and establish symptoms, which was not necessary as these were established at the baseline session. Therefore, the first therapy session combined a brief introduction to MCT+ (Unit 2), generating an illness model (Unit 3), and covered attributional styles (Unit 4), which specifically observed the importance of considering multiple attributions (e.g., situational, personal, internal) jointly for a single event. The second therapy session combined Unit 5 on decision-making, which looks at the jumping to conclusions (JTC) bias and the importance of gathering sufficient evidence before making a decision, and Unit 6 on changing beliefs, which encourages clients to re-evaluate the validity of their opinions and change them when necessary, rather than always insisting on one's opinion and/or ignoring disconfirming evidence. The third session covered Unit 7 on empathizing (e.g., the complexity of social cues and the importance of collecting multiple social cues before making strong social inferences) and Unit 8 on overconfidence in memory errors. The final therapy session focused on improving self-esteem and mood by looking that factors that perpetuate depressive styles of thinking (Unit 9), and concluded by looking at relapse prevention (Unit 10). For an in-depth description of all MCT+ therapy units, please refer to Balzan et al. (2014b) or by way of the following link: http://www.clinical-neuropsychology.de/metacognitive-therapy-plus-individualized-mct-for-psychosis.html.

### Baseline and follow-up assessment

A number of assessments observing clinical and cognitive domains were made as part of the larger trial. Only assessments pertaining to the current case study are documented here. As the principle aim of MCT+ is reduce the severity of delusional ideation, a number of measures were included to assess delusional propensity. Interview-led measures of delusional severity included the Positive and Negative Syndrome Scale (PANSS; Kay et al., 1987), which consists of seven positive, seven negative, and 16 general psychotic symptoms; the Psychotic Symptom Rating Scales (PSYRATS; Haddock et al., 1999), which focuses on the frequency and duration of hallucination and delusions; and the Scale for the Assessment of Positive Symptoms (SAPS; Andreasen and Olsen, 1982), which covers a variety of common hallucinatory and delusional themes (e.g., auditory hallucinations, delusions of reference, persecutory delusions). Clinical insight was estimated using the Schedule for Assessing Insight (SAI) for psychosis patients (adapted from David, 1990). The PANSS and PSYRATS interview-based assessments were undertaken by a trained rater blind to treatment allocation (i.e., this rater did not conduct the intervention). Additionally, clients completed the self-report 21-item Peters et al Delusions Inventory (PDI-21; Peters et al., 2004), which provides a scale for global delusional ideation, and subscales for delusional distress, preoccupation and conviction. Pre-morbid intelligence was estimated by the Wechsler Test of Adult Reading (WTAR; Wechsler, 2001).

The “illusion of control” bias, shown to be higher in delusional samples (Balzan et al., 2013b), was assessed at baseline and follow-up, consistent with a previous MCT+ efficacy study that observed changes in this bias post-intervention (Balzan et al., 2014a)^1^. Illusory control was assessed using a non-contingent “tone task” adapted from Matute (1995). Participants were presented with four buttons (labeled A, B, C, and D) on a screen that could be activated using a mouse click, and instructed that they would periodically hear a loud “tone” noise (maximum duration 5-s), and that their task was to find a way to stop it within this time by clicking the correct combination of the four A, B, C, and D buttons. The task comprised 40 trials of uncontrollable tones (i.e., all tones were non-contingent on the participant's response); 75% of tones terminated automatically after 1-s (i.e., 30 trials appeared to turn off after clicking buttons), and 25% terminated after 5-s (i.e., 10 trials appeared to “max out”). After the 40 trials, participants were asked to indicate the percentage of control they had over the termination of tones, and the percentage of trials in which the tones terminated because they had clicked on the correct sequence of buttons (i.e., perceived response-outcome connection).

## Results

Table 1 summarizes the baseline and follow-up scores across the clinical and cognitive measures of interest for both clients. Symptom severity was reduced across all measures used. The overall reductions in PANSS scores (Client 1: −9 points; Client 2: −19 points) were reflected in the positive subscale (Client 1: −5 points; Client 2: −7 points); importantly, there was modest reduction in the delusions item (P1) specifically (Client 1: −1 point; Client 2: −2 points). This reduction in delusional severity was mirrored by the both the PSYRATS (Client 1: −9 points; Client 2: −19 points) and SAPS (Client 1: −7 points; Client 2: −5 points) delusions subscales, which take into account the frequency, duration, distress, and level of conviction of the delusional belief/s. Interestingly, both clients self-reported reductions in delusional distress, preoccupation, and conviction as evidenced by the PDI scale (Client 1: −38 points; Client 2: −25 points).

Clinical insight was minimal in both clients at baseline (i.e., Client 1 scored 2 and Client 2 scored 8, out of a maximum score of 18) but improved post-intervention, whereby both clients started to doubt the validity/creditability of their beliefs, and admitted the cause of their unusual experiences may be due to internal causes (e.g., stress) rather than purely delusional causes (e.g., chip inserted into neck). Client 1 also acknowledged the potential role of THC in heightening the severity of his paranoia, and was open to cutting down his usage, and even resuming antipsychotic medication. Improvements were also observed for the illusory control bias task, whereby both clients expressed reduced perceived control over a non-contingent task (Client 2 correctly responded zero control at follow-up), and less perceived “response-outcome” connection.

## Discussion

The present case study reports the impact of individualized metacognitive therapy (MCT+) in two clients with psychosis (schizophrenia and delusional disorder), who were experiencing active delusions, but were not receiving antipsychotic medication at the time of the current trial. MCT+ aims to improve the well-being of people living with psychosis, with a particular focus on reducing the severity of delusional symptoms. MCT+ achieves this by (1) bringing about an awareness of the underlying cognitive biases or “traps” that are thought to contribute the formation and maintenance of delusions, (2) offering clients strategies to reduce their propensity to these biases, and (3) relating the material to the personal experiences and belief systems of the individual client.

The current findings suggest that MCT+ is effective in reducing the symptoms of psychosis, and notably delusional ideation, in the absence of antipsychotic medication. For both clients, we observed overall improvements in positive symptoms and delusional conviction, preoccupation, frequency, and level of distress they caused (assessed by blind interviewer and self-report). Clinical insight was still low at follow-up, but had improved from baseline, and propensity to the illusion of control bias was reduced. Of note, the illusion of control bias is not specifically targeted in any of the MCT modules, which suggests that MCT may be improving some underlying cognitive mechanism that is responsible for a variety of cognitive biases observed in psychosis (Balzan et al., 2014a). It is also worth pointing out that neither client missed a single session (i.e., all nine therapy units were covered over the 4-week therapy phase), which highlights the ability of the therapy program to motivate and actively engage with clients (even those with minimal clinical insight), without being too confrontational or damaging to the therapeutic alliance. The results also demonstrate that the therapy program may be effective across multiple diagnoses (i.e., the majority of MCT studies to date have mainly observed schizophrenia or schizoaffective disorders), and different levels of functioning (i.e., Client 2 had higher “above-average” pre-morbid intelligence).

Overall, these findings are not only consistent with the growing evidence-base for MCT (e.g., Moritz et al., 2014a), but are also consistent with recent findings suggesting that psychotherapy may be effective even in the absence of antipsychotic medication or in treatment-resistant clients (Morrison et al., 2014). Although the results of this case study are promising, a number of methodological issues, common to the majority of case studies, should be acknowledged. First, the results should not be broadly generalized, and the reported improvements may actually represent statistically non-significant trends in the larger participant sample. Further hindering generalization of the results is the lack of extended (e.g., 6-month) follow-up data, which would provide evidence on the sustainability of the reported improvements, and the SAPS assessment was made by a rater who was aware of group allocation. Neither client exhibited the typical JTC bias at baseline (i.e., definite decision on two or less beads), which ruled out the possibility of observing a reduction in JTC post-intervention. Moreover, it is possible that the observed improvements may be attributable to the natural fluctuations of psychotic symptoms (“waxing and waning”) that occur across time, or to practice effects in the illusion of control task. The results could also reflect a more general effect of the therapeutic relationship. More methodologically rigorous randomized control trials evaluating the efficacy of MCT+ are required to properly address these issues.

## Concluding remarks

Psychotherapeutic approaches in the treatment of psychosis have been gaining ground in recent years, and have been shown to be effective as adjunctive therapy when used alongside antipsychotic medication, and represent a better treatment option when added to antipsychotic therapy, than pharmacological therapy alone. The therapeutic efficacy of psychotherapy in “treatment-resistant” clients or where antipsychotic adherence is poor, is much less well-understood. The current case study suggests that individualized metacognitive therapy (MCT+), a combination of the “process-oriented” approach of the MCT group-training and individual cognitive-behavioral therapy for psychosis (CBTp), may be an effective treatment option in such cases.

### Conflict of interest statement

The authors declare that the research was conducted in the absence of any commercial or financial relationships that could be construed as a potential conflict of interest.

## Footnotes

^1^Note: JTC was also assessed using the “beads task” (for a detailed overview, see Fine et al., 2007), but neither client exhibited a “JTC bias” at baseline (i.e., definite decision on first or second bead), so results of this task are not reported.
